# Spatiotemporal Contextual Uncertainties in Green Space Exposure Measures: Exploring a Time Series of the Normalized Difference Vegetation Indices

**DOI:** 10.3390/ijerph16050852

**Published:** 2019-03-08

**Authors:** Marco Helbich

**Affiliations:** Department of Human Geography and Spatial Planning, Faculty of Geosciences, Utrecht University, Princetonlaan 8a, 3584 CB Utrecht, The Netherlands; m.helbich@uu.nl; Tel.: +31-30-253-2017

**Keywords:** health, green space, vegetation indices, NDVI, MODIS, exposure, spatial and temporal contextual uncertainties, NEEDS study

## Abstract

Environmental health studies on green space may be affected by contextual uncertainties originating from the temporality of environmental exposures and by how the spatial context is delimitated. The Normalized Difference Vegetation Index (NDVI) is frequently used as an outdoor green space metric capturing the chlorophyll content in the vegetation canopy. This study assessed (1) whether residential NDVI exposures vary over time, and (2) how these time series of NDVI scores vary across spatial context delimitations. Multi-temporal NDVI data for the period 2006–2017 for the Netherlands were obtained from the Moderate Resolution Imaging Spectroradiometer (MODIS) satellite platform. Annual NDVI exposures were determined across multiple buffer sizes (i.e., 300, 600, and 1000 m) centered on a random sample of 10,000 Dutch residential addresses. Besides the descriptive statistics, pairwise Wilcoxon tests and Fligner–Killeen tests were used to determine mean and variance differences in annual NDVI scores across buffer widths. Heat maps visualized the correlation matrices. Significance levels were adjusted for multiple hypotheses testing. The results indicated that annual NDVI metrics were significantly correlated but their magnitude varied notably between 0.60 to 0.97. Numerous mean and variance differences in annual NDVI exposures were significant. It seems that the disparate buffers (i.e., 300 and 1000 m) were less strongly correlated, possibly because variance heterogeneity is reduced in larger buffers. These results have been largely consistent over the years and have passed Monte Carlo-based sensitivity tests. In conclusion, besides assessing green space exposures along different buffer sizes, our findings suggest that green space–health studies should employ NDVI data that are well-aligned with epidemiological data. Even an annual temporal incompatibility may obscure or distort green space–health associations. Both strategies may diminish contextual uncertainties in environmental exposure assessments.

## 1. Introduction

It is increasingly recognized that the human body responds to the environments to which it is exposed [1,2,3]. The assessment of health-threatening and health-promoting environmental exposures has received fresh impetus from progress in earth observation coupled with geographic information systems (GIS) leading to high-quality environmental information [4,5]. To date, satellite images represent the gold standard for capturing conditions of the natural environment (i.e., vegetation) in an objective manner [5,6,7].

One prominent environmental factor is green space—namely forests, parks, etc.—which is capable of reducing harm caused by exposure to physical and mental health stressors [2,8]. Studies found that being surrounded by outdoor residential green space has beneficial effects for diverse population groups and on multiple health outcomes, such as adiposity [9], birth outcomes [10], suicide [11], physical activity [12], children’s brain development and cognitive function [13], the elderly’s mortality [14], and depression [15]. Pathways connecting green space and health include the mitigation of harmful exposures (e.g., air pollution, noise), the promotion of physical activity, and the support of restoring capacities [8].

Vegetation metrics [7] derived from remote sensing images such as the Normalized Difference Vegetation Index (NDVI) [16,17] are widely used to describe the distribution and amount of green space in population-based epidemiological studies [14,18,19]. However, findings that relate NDVI to health outcomes are not always consistent. For example, while [13] reported beneficial effects of green space on brain development, another study found an insignificant direct effect of residential NDVI extracted from the Moderate Resolution Imaging Spectroradiometer (MODIS) on major depressive disorders and psychological distress [20].

While there are manifold reasons for these contradictory findings (e.g., different research designs, study site specific differences), contextual uncertainties also contribute, to a certain extent, to the heterogeneity of findings on green space and health outcomes [21]. First, the temporality of environmental exposures contributes to contextual uncertainties. Green space varies naturally over time, but ambiguities can also arise artificially as a result of applied data processing [22]. Despite such temporal variations in NDVI scores, some studies [20,23] defend the application of NDVI data that are misaligned with epidemiological data. However, empirical results supporting such practices are lacking. Since longitudinal studies incorporating people’s life courses and their exposure to (temporally weighted) time series of green space are receiving increasing attention [9,24], well-aligned time series of NDVI data are crucial to circumvent exposure misclassifications.

Spatial contextual uncertainties arise due to the diverse ways in which the spatial context that people belong to is defined. Several studies have shown that assigning exposures based on administrative areas (e.g., postal codes) leads to conceptual and methodological flaws (for details, see [3]). Refined green space assessments can be achieved by focusing only on the vicinity of an individual’s actual residential location as represented through GIS-based buffers. As the “true” health influencing spatial context is unknown, the ways to determine an adequate buffer size are still under debate. Consensus has been reached, however, that different buffers sizes need to be explored to validate the stability of health–NDVI associations [25,26].

Motivated by these challenges, this study addressed spatiotemporal contextual uncertainties in exposure to outdoor NDVI for a large sample of addresses in the Netherlands. The first objective was to examine whether NDVI exposure measures vary significantly over time. Such analyses empirically underpin common empirical practices whereby temporally misaligned indicators are used as substitutes in assessing health–green space correlations. The second objective was to examine how variations in the spatial context—here defined as buffers with different sizes centered on residential addresses—lead to temporally misaligned exposure measures.

The empirical findings contribute to a better understanding of contextual uncertainties about NDVI scores. As spatial and temporal contextual uncertainties jointly affect green space assessments, this study puts both scale issues and the temporal alignment of green space exposures centrally. The results may assist those carrying out future studies to decide whether misaligned vegetation indices could serve as substitutes. To the best of our knowledge, no previous study has addressed these elementary but important issues, which have the potential to result in biased green space exposure assessments.

## 2. Materials and Methods

### 2.1. Study Area

This analysis was conducted in the Netherlands as part of the NEEDS study (www.needs.sites.uu.nl). The Netherlands was selected as a case study due to its diverse geography, which ranges from rural areas with much green space in the north of the country, to one of Europe’s most populated and urbanized areas in the middle of the country (i.e., the Randstad area).

### 2.2. Address Data

**Figure A1:**
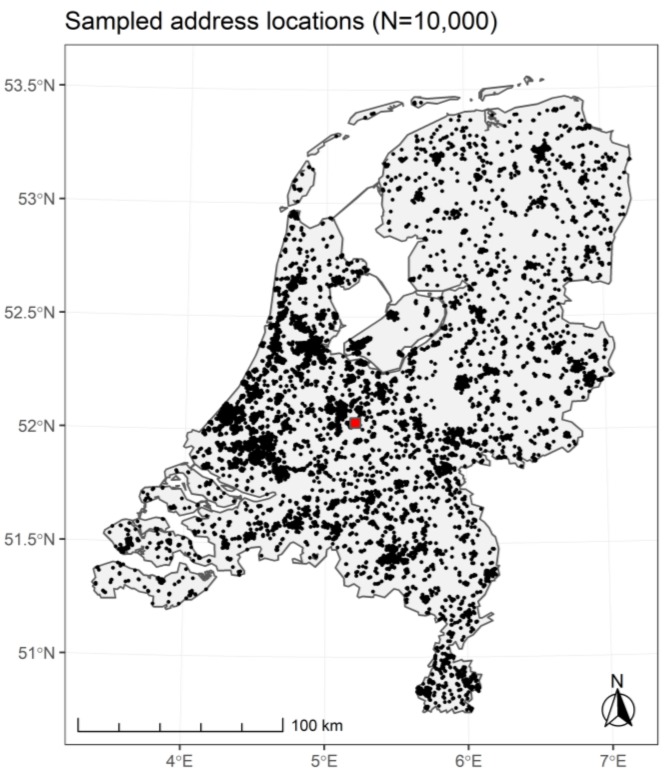
Sampled address locations. The red dot in the center indicates the location shown in Figure 1.

Like previous studies [23,27], the analysis was carried out at an address level. Address locations stem from the Dutch cadaster (version September 2017), which contains 9.1 million addresses. We extracted those for residential function and mixed use. From these 8.7 million addresses, a random sample of 10,000 locations was drawn; each had the same selection probability. Such a sample size corresponds to epidemiological studies. Figure A1 in the Appendix shows the distribution of the sampled addresses.

### 2.3. Remote Sensing-Based NDVI Data

The widely implemented satellite-derived Normalized Difference Vegetation Index (NDVI) was used to measure the level of greenness [17]. Such a multispectral vegetation index reflects the fact that chlorophyll absorbs the red light (i.e., 0.6–0.7 µm) and the mesophyll leaf structure scatters the near-infrared (0.7–1.1 µm) [16,28,29]. The NDVI is composed of the near-infrared radiation minus visible radiation divided by near-infrared radiation plus visible radiation [17]. The NDVI ranges between −1 and +1, where negative values refer to non-biomass (e.g., clouds, water, and snow) and values around 0 represent rocks and bare soil. The higher the positive values, the higher the amounts of biomass. To reduce the likelihood of biased exposure determination, values <0 (representing water bodies) are filtered for outdoor green space assessments [23].

**Figure 1:**
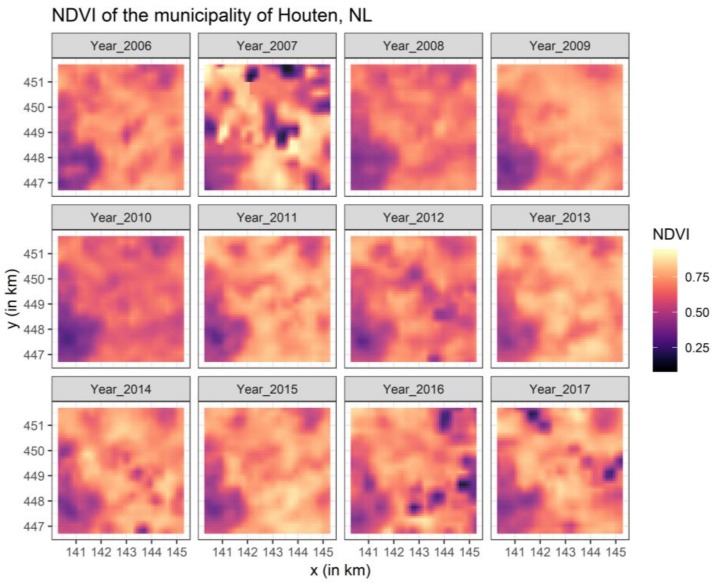
Example of the annual NDVI time series from 2006–2017 for the municipality of Houten, the Netherlands.

We focused on MODIS NDVI satellite-based measures due to their global coverage as pre-processed time series while being less computationally intensive than Landsat or other higher resolution data. Annual MODIS NDVI 16-day composites (June 25–July 10, with the product ID MOD13Q1) in the hierarchical data format were acquired for the Netherlands spanning the period 2006–2017 [7,29]. Within the 16-day acquisition period, the algorithm to pre-process the MODIS data selected pixels with the lowest cloud coverage, a low view angle, as well as having the highest NDVI values [29]. Data were obtained through the online MODIS data repository (https://modis.gsfc.nasa.gov/data/dataprod/mod13.php). As vegetation follows a seasonal cycle with the highest amount of green during the summer, we consistently used annual data for June/July. To match with other available data, the original spatial resolution of 250 meters was down-scaled to 100 meters using bilinear interpolation. After clipping and re-projecting the data to the national Dutch reference system, the data were entered into a multilayer image stack, and the recommended scaling factor of 0.0001 was applied [29]. Figure 1 illustrates an example of the NDVI time series for Houten and its surroundings.

### 2.4. GIS-Based Exposure Assessment

To represent the spatial environmental context, we utilized a buffer approach using GIS [25,27]. Circular buffers with radii of 300, 600, and 1000 m were centered on each sampled address location. Smaller buffer sizes reflect the immediate surroundings, while 1000 m buffers capture the wider surroundings. In addition, such circular buffers allow for the exploration of scale effects and are more suitable for long-term analyses because they are independent of auxiliary data (i.e., the road network), which also change over time but are rarely available. Next, we determined the average NDVI values for every address across the three buffer sizes for each year. These proxy measures for green space exposure are attached to each location.

### 2.5. Statistical Analyses

Methods included descriptive statistics describing the central tendency including the mean, standard deviation, etc., to summarize the residential NDVI scores. Individual NDVI trajectories for each address and year were displayed through time series plots. Non-parametric correlations after Spearman coupled with a heat map [30] were implemented to assess associations across the different buffer sizes as well as their correlations over time. Pairwise Wilcoxon rank sum tests were used to test whether NDVI scores differ statistically over the years and across different buffer sizes [31]. Non-parametric Fligner–Killeen tests, which are robust against non-normally distributed values, were conducted to test whether the variances across the annual NDVI scores and buffer sizes differ [32].

Since we conducted a large number of tests, the multiple testing problem arose, namely the probability of false positives increased (i.e., finding differences by chance). To handle multiple hypotheses testing in the applied statistics, the *p*-values were adjusted following Holm’s procedure [33]. Finally, because the sample characteristics could vary by chance, sensitivity tests were performed. A Monte Carlo procedure [34] was used to examine whether the initial sample mean varied significantly compared to 100 alternative random samples, each 1000 addresses large, drawn from all residential addresses (i.e., the initial 10,000 addresses were excluded). The annual mean and standard deviation (SD) of the resulting reference distribution was compared to the initial sample per year. The analyses were carried out in the R environment for statistical computing [35].

## 3. Results

**Figure 2:**
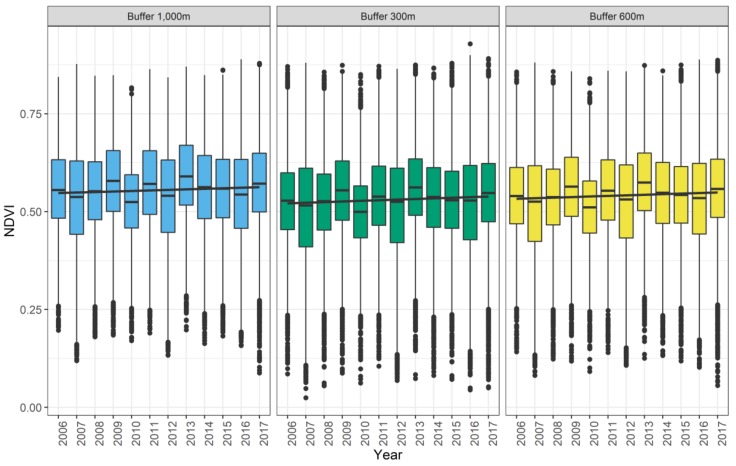
Boxplots of the NDVI scores per year across different buffer sizes. The black regression line refers to the trend over time.

Table 1 together with Figure 2 summarize the annual NDVI scores aggregated across the sample. The boxplots confirm what the measures of central tendency indicated, namely that the NDVI values fluctuate slightly between 2006 and 2017. For example, the NDVI values for 2010 seem to be lower compared to the other years. There seems to be a minor overall temporal trend of increasing NDVI scores since 2006. Regression lines added to the boxplots confirm this impression. Similar patterns were observed when cross-comparing the different buffer sizes.

**Figure 3:**
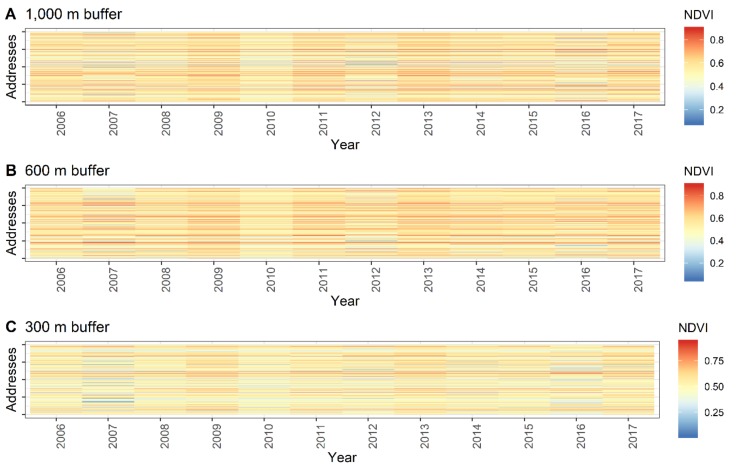
NDVI time series for the sampled addresses using different buffer radii. Each horizontal line refers to the NDVI trend of an address (*N* = 10,000).

Because such aggregated analyses hide individual temporal patterns, the individual time series of the average 16-days NDVI composite values per address location were also examined. Figure 3 displays the time series plots. While the overall plots do not indicate striking patterns, an individual investigation showed some striking temporal changes of the NDVI scores per location.

**Figure 4:**
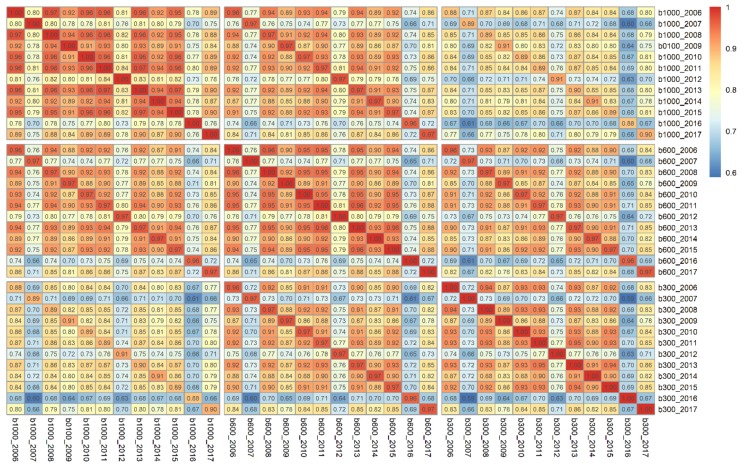
Heat map of the correlations for the NDVI time series for the 1000, 600, and 300 m buffers. All plotted correlation values were significant after adjusting for multiple testing.

The core of the analyses was, as summarized in Figure 4, assessing the NDVI correlations over time and across buffer sizes. While all correlations were highly statistically significant (*p* < 0.05), even after adjusting for multiple testing, some patterns were recognizable. For example, the 1000 m buffers were less strongly correlated than the 600 and 300 m buffers. Further, it seemed that some years were less associated, although still statistically significant. The years 2007 and 2016 were, independent of the examined buffer size, less associated with the other years of NDVI data. This was in line with Figure 1, whereas deviations in the NDVI patterns were recognizable. Even annual differences of a single year may result in some correlation differences.

**Figure A2:**
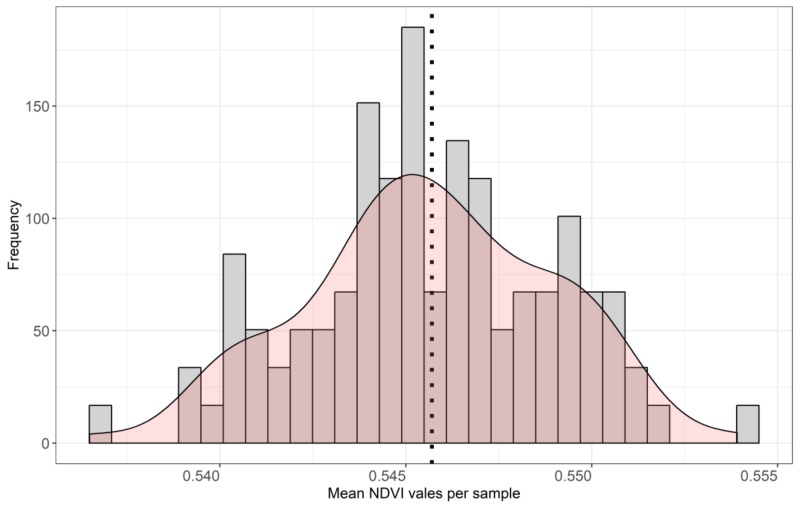
Simulated reference distribution of mean NDVI values for 2014 using a 600 m buffer based on 100 samples each 1000 comprising addresses. The dotted line refers to the mean NDVI value of the initial sample.

The results of the pairwise mean comparisons between years with corrections for multiple testing are given in Table 2. As indicated by the *p*-values, several years of our sampled NDVI values have different means, independent of which buffer size is considered. Moreover, the Fligner–Killeen tests to assess the homogeneity of variances rejected the null hypothesis of equal variances over the years and buffer sizes (all *p* < 0.001). Our final sensitivity tests, using 100 random samples of 1000 address samples and comparing mean NDVI scores, indicated no statistically significant differences. The initial mean NDVI score was well-situated in the simulated distribution of mean NDVI values plus/minus one standard deviation. Figure A2 in the Appendix illustrates the result for NDVI values for 2014 and the 600 m buffer.

## 4. Discussion

Accurately assessing the level of outdoor green space surrounding people’s residential homes is crucial for epidemiological studies. Due to its cost-effectivity and large coverage of high-quality time series, the NDVI is commonly used for regional or even nationwide studies [14,19]. To limit contextual uncertainties—a fundamental methodological problem [21]—it is important to have NDVI data that are temporally well-aligned with the epidemiological data, although it is not common practice [20,23]. Such data mismatch may translate into biased outcomes, that is, lead to an over- or underestimation of the NDVI effect size in regressions. This study shed light on the often ignored circumstance of temporally incompatible green space data by exploring annual time series of NDVI exposures derived from buffers centered on a large sample of residential addresses randomly distributed across the Netherlands.

### 4.1. Interpretation of the Results

The results showed that, as expected, individual years of NDVI scores were significantly correlated; however, a few years were less strongly correlated than others. These differences were further confirmed statistically through mean and variance differences in NDVI exposures. Our findings question the practice of having a temporally poorly-aligned linkage of green space and epidemiological data. For example, Rugel et al. [20] used survey data for 2012 while the NDVI was for 2014–2016. At least partly due to temporally incompatible data, an insignificant green space effect on mental health outcomes was reported by that study. It could be that the level of green space changed, which translated into less accurate exposure assessments. However, the NDVI differences could also emerge due to mixed pixels, misregistration of the satellite location, etc. (see [16] for a discussion).

There is no consensus on the buffer size to delineate the residential context [25,26]. Our tested buffer widths followed the literature, reflecting the closer and extended residential environment. The results show that the NDVIs for different buffer widths over time are highly positively correlated, ranging from 0.60 to 0.97. A similar range of correlations for MODIS NDVIs was reported elsewhere [26]. Furthermore, there seems to be a tendency for the disparate buffers to be less correlated, which is in concordance with a study in New York City [26]. An explanation could be that smaller buffers have a pronounced variance while NDVI differences average out in larger buffers. Roughly, these results are consistent over the time series of NDVI values. Utilizing a Monte Carlo approach, these results passed sensitivity tests. It can be ruled out that the initial sample represented an extreme case. Given these results, it is recommended not to use temporally incompatible NDVI and epidemiological survey data. To reduce temporal uncertainties, the date should be aligned as closely as possible with the survey data. Without having conducted the obligatory tests, we speculate that discrepancies may be attenuated when cumulative green space exposures over time (or time-weighted ones), rather than purely cross-sectional metrics, are employed. Here, systematic investigations across different study designs, health outcomes, study sites, etc. require further attention.

Because MODIS NDVI maps are retrieved from daily images to generate 16-day composite measures, exact temporal matches are possible for most parts of the world, which is challenging for other popular sensor platforms such as Landsat. The promising temporal granularity of MODIS images is challenged by a moderate spatial resolution of 250 meters and a minimum mapping size of detectable vegetation. This may be problematic for urban areas where small-scale green spaces exist, which may lead to an over- or underestimation of the true green space exposure. Besides these technological constraints, it is debatable whether green space measures from downward-facing satellites (including all platforms) represent accurately how people on the ground perceive green space [36,37]. When comparing neighborhood-based NDVI scores and street view-based green space for Beijing (China), no significant associations were found across both metrics [37]. Such overhead-view assessments only capture the available vegetation; they do not provide insights into the quality, accessibility, and actual use of these areas [20,38].

The application of green space metrics is more complex than it seems at first sight due not only to different sensor platforms (e.g., MODIS, Landsat) but also to complications ensuing from the variety of measures proposed. While the NDVI correlates with expert ratings [39], the enhanced vegetation index (i.e., an alternative vegetation index), for example, is designed to overcome methodological limitations of the NDVI including the elimination of atmospheric and canopy background effects contaminating standard NDVI while having better sensitivity at higher vegetation levels [7,29] (for an application, see [20]). Others implemented classified land use information [15,27,40]—which is challenging because of a varying number of land use categories or sensitivity to the underlying image-based classification algorithm—or used measures such as the leaf area index [41] and the vegetation continuous fields tree cover [42,43]. However, our results are of more general interest because the MODIS NDVI was found to be highly correlated with alternative vegetation measures such as the leaf area index [41].

### 4.2. Study Limitations

There are numerous limitations to this analysis. Firstly, the present analytical study design is restricted. While imitating real-word ego-centric exposure assessments, the study did not evaluate the actual consequences of misaligned green space measures on physical and mental health outcomes across different settings (e.g., countries, cities and rural areas, spatial contexts). Studies that rule out incompatible green space data and that do not lead to biased regression coefficients are advised. Secondly, although down-scaling increased the number of cells per buffer while limiting the influence of edge effects, we cannot exclude that this procedure artificially over- or underestimated the average NDVI values. Furthermore, when interpreting the results, it is critical to stress that all NDVI values given, within the threshold buffer distance, contribute equally (i.e., mean of NDVI cells per buffer). A refinement could be distance-based weighting schemes because cells close to homes are more important than those further away. Thirdly, notwithstanding that NDVI was found to be fairly constant across satellite platforms [6], only MODIS NDVI measures were explored. Thus, the results should not be uncritically transferred to other platforms (e.g., RapidEye, Sentinel 2). Such high-resolution sensors support more precise inner-urban analyses. Fourthly, for the sake of simplicity, we assumed the residential home location to be the source of exposure, as did others [19,23]. This conventional conceptualization leaves aside exposures along people’s day-to-day mobility, which is relevant for more accurate exposure assessments [3,21]. Finally, as only a large, representative sample for the Netherlands was investigated, the generalization of findings could be questioned.

## 5. Conclusions

This paper addressed contextual uncertainties originating from both the temporality of environmental exposures and how the spatial context is delimitated. The empirical results show that annual NDVI scores derived from different buffer sizes differed significantly in their means and variances. Furthermore, within the range of 0.60 to 0.97, the magnitude of the correlations varied notably over the years and buffer sizes.

These findings are vital for exposure assessments that often enrich epidemiological data with temporally incompatible NDVI data, thus potentially obscuring green space–health associations. Even a misalignment of a single year may distort correlations. To mitigate contextual uncertainties, it is advised to integrate temporally well-aligned green space data. While the present study took a first step toward an empirical basis addressing spatiotemporal contextual uncertainties, future studies should systematically research the consequences for correlation studies.

## Figures and Tables

**Table 1:** Summary statistics for the residential NDVI scores (*N* = 10,000).

Buffer (m)	Year	Mean	SD	Median	25th Percentile	75th Percentile	Min.	Max	Skewness	Kurtosis
1000	2006	0.55	0.11	0.56	0.48	0.63	0.197	0.84	−0.1595	−0.2427
	2007	0.53	0.14	0.54	0.44	0.63	0.119	0.88	−0.3160	−0.1868
	2008	0.55	0.11	0.55	0.48	0.63	0.180	0.85	−0.2441	−0.0829
	2009	0.57	0.11	0.58	0.50	0.66	0.185	0.85	−0.2540	−0.2093
	2010	0.52	0.10	0.52	0.46	0.59	0.170	0.82	−0.1243	−0.1466
	2011	0.57	0.12	0.57	0.49	0.66	0.190	0.86	−0.1028	−0.3292
	2012	0.53	0.13	0.54	0.45	0.63	0.133	0.84	−0.3213	−0.2992
	2013	0.59	0.11	0.59	0.52	0.67	0.198	0.87	−0.2091	−0.2376
	2014	0.56	0.12	0.56	0.48	0.64	0.163	0.85	−0.2075	−0.2629
	2015	0.56	0.11	0.56	0.48	0.63	0.182	0.86	−0.1228	−0.1823
	2016	0.54	0.13	0.54	0.46	0.63	0.158	0.89	−0.1377	−0.3493
	2017	0.57	0.12	0.57	0.50	0.65	0.088	0.88	−0.3084	0.1690
600	2006	0.54	0.11	0.54	0.47	0.61	0.142	0.86	−0.1232	−0.0792
	2007	0.52	0.14	0.53	0.42	0.62	0.082	0.88	−0.2706	−0.2122
	2008	0.54	0.11	0.54	0.47	0.61	0.123	0.86	−0.2067	0.1153
	2009	0.56	0.11	0.56	0.49	0.64	0.118	0.86	−0.2223	−0.0631
	2010	0.51	0.10	0.51	0.45	0.58	0.092	0.84	−0.0708	0.0195
	2011	0.56	0.12	0.55	0.48	0.63	0.140	0.86	−0.0320	−0.1048
	2012	0.52	0.13	0.53	0.43	0.62	0.107	0.86	−0.3209	−0.2185
	2013	0.57	0.11	0.57	0.50	0.65	0.126	0.87	−0.1449	−0.0414
	2014	0.55	0.12	0.55	0.47	0.63	0.132	0.86	−0.1682	−0.0987
	2015	0.54	0.11	0.54	0.47	0.62	0.119	0.87	−0.0627	0.0493
	2016	0.53	0.13	0.53	0.44	0.62	0.102	0.89	−0.1643	−0.2389
	2017	0.56	0.12	0.56	0.49	0.63	0.056	0.89	−0.3125	0.4280
300	2006	0.53	0.11	0.53	0.45	0.60	0.086	0.87	−0.0937	0.0042
	2007	0.51	0.15	0.52	0.41	0.61	0.024	0.88	−0.2453	−0.2379
	2008	0.52	0.11	0.53	0.45	0.60	0.055	0.86	−0.1837	0.2192
	2009	0.55	0.12	0.55	0.48	0.63	0.087	0.87	−0.2108	0.0401
	2010	0.50	0.10	0.50	0.43	0.57	0.062	0.85	−0.0209	0.1092
	2011	0.54	0.12	0.54	0.47	0.62	0.105	0.87	0.0241	0.0100
	2012	0.51	0.14	0.53	0.42	0.61	0.069	0.86	−0.3458	−0.1663
	2013	0.56	0.11	0.56	0.49	0.63	0.074	0.87	−0.0903	0.1060
	2014	0.53	0.12	0.54	0.46	0.61	0.082	0.87	−0.1569	0.0586
	2015	0.53	0.11	0.53	0.46	0.60	0.072	0.88	−0.0047	0.1354
	2016	0.52	0.14	0.53	0.43	0.62	0.045	0.93	−0.2037	−0.1898
	2017	0.55	0.12	0.55	0.47	0.62	0.050	0.89	−0.3184	0.5647

**Table 2:** Significance levels for the Wilcoxon tests (*p*-values adjusted after Holm).

Buffer (m)	Year	2006	2007	2008	2009	2010	2011	2012	2013	2014	2015	2016
1000	2007	0										
	2008	0.034	0									
	2009	0	0	0								
	2010	0	0	0	0							
	2011	0	0	0	0.067	0						
	2012	0	0.331	0	0	0	0					
	2013	0	0	0	0	0	0	0				
	2014	0.006	0	0	0	0	0	0	0			
	2015	0.331	0	0	0	0	0	0	0	0.331		
	2016	0	0	0	0	0	0	0.065	0	0	0	
	2017	0	0	0	0.023	0	0.708	0	0	0	0	0
600	2007	0										
	2008	0.132	0									
	2009	0	0	0								
	2010	0	0	0	0							
	2011	0	0	0	0	0						
	2012	0	0.132	0	0	0	0					
	2013	0	0	0	0	0	0	0				
	2014	0	0	0	0	0	0	0	0			
	2015	0.132	0	0	0	0	0	0	0	0.064		
	2016	0.001	0	0.132	0	0	0	0.015	0	0	0	
	2017	0	0	0	0.008	0	0.132	0	0	0	0	0
300	2007	0										
	2008	0.437	0									
	2009	0	0	0								
	2010	0	0	0	0							
	2011	0	0	0	0	0						
	2012	0	0.058	0.027	0	0	0					
	2013	0	0	0	0	0	0	0				
	2014	0	0	0	0	0	0.067	0	0			
	2015	0.181	0	0.006	0	0	0	0	0	0.004		
	2016	0.68	0	0.68	0	0	0	0.009	0	0	0.027	
	2017	0	0	0	0.005	0	0	0	0	0	0	0
